# Depression sum-scores don’t add up: why analyzing specific depression symptoms is essential

**DOI:** 10.1186/s12916-015-0325-4

**Published:** 2015-04-06

**Authors:** Eiko I Fried, Randolph M Nesse

**Affiliations:** 1 grid.5596.f0000000106687884University of Leuven, Faculty of Psychology and Educational Sciences, Research Group of Quantitative Psychology and Individual Differences, Tiensestraat 102, 3000 Leuven, Belgium; 2 grid.215654.10000000121512636School of Life Sciences, Arizona State University, Room 351 Life Sciences Building A, Tempe, AZ 85287-450 USA

**Keywords:** Depression symptoms, Diagnostic and Statistical Manual of Mental Disorders, Heterogeneity, Major depressive disorder, Nosology

## Abstract

Most measures of depression severity are based on the number of reported symptoms, and threshold scores are often used to classify individuals as healthy or depressed. This method – and research results based on it – are valid if depression is a single condition, and all symptoms are equally good severity indicators. Here, we review a host of studies documenting that specific depressive symptoms like sad mood, insomnia, concentration problems, and suicidal ideation are distinct phenomena that differ from each other in important dimensions such as underlying biology, impact on impairment, and risk factors. Furthermore, specific life events predict increases in particular depression symptoms, and there is evidence for direct causal links among symptoms. We suggest that the pervasive use of sum-scores to estimate depression severity has obfuscated crucial insights and contributed to the lack of progress in key research areas such as identifying biomarkers and more efficacious antidepressants. The analysis of individual symptoms and their causal associations offers a way forward. We offer specific suggestions with practical implications for future research.

**Electronic supplementary material:**

The online version of this article (doi:10.1186/s12916-015-0325-4) contains supplementary material, which is available to authorized users.

## Background

“*At present major depression has become a monolith, with the assumption that the diagnosis can be made merely on the number of depressive symptoms present* […]*. It may be politically important to utter such simplifications to doctors in general medical settings, but it is a convenient fiction.*”

– Goldberg, 2011, p. 227 [1]

Major depressive disorder (MDD) is one of the most common psychiatric disorders, with an estimated lifetime prevalence rate in the USA of 16.2% [2]. It is the leading cause of disability worldwide, and one of the top three causes of disease burden worldwide [3]. About 60% of individuals meeting criteria for MDD, as defined by the Diagnostic and Statistical Manual of Mental Disorders (DSM-5) [4], report severe or very severe impairment of functioning [2] that highly compromises the capacity for self-care and independent living.

The severity of MDD is routinely estimated by adding up severity scores for many disparate symptoms to create a sum-score, and threshold values for these sum-scores are commonly used to classify individuals as depressed or not depressed. This practice of constructing sum-scores and collapsing individuals with different symptoms into one undifferentiated category is based on the assumption that depression is a single condition, and that all symptoms are interchangeable and equally good indicators. This review shows that this common practice discards much critical information about individual symptoms whose analysis can provide important insights.

### Depression heterogeneity

In the DSM-5, MDD is characterized by nine symptoms: 1. depressed mood; 2. markedly diminished interest or pleasure; 3. increase or decrease in either weight or appetite; 4. insomnia or hypersomnia; 5. psychomotor agitation or retardation; 6. fatigue or loss of energy; 7. feelings of worthlessness or inappropriate guilt; 8. diminished ability to think or concentrate, or indecisiveness; and 9. recurrent thoughts of death or recurrent suicidal ideation. To qualify for the diagnosis, an individual must exhibit five or more symptoms, one of which must be either depressed mood or anhedonia. Of note, all symptoms except the first contain sub-symptoms (e.g., diminished interest or pleasure). Moreover, three symptoms – sleep problems, weight/appetite problems, and psychomotor problems – encompass opposite features (insomnia vs. hypersomnia; weight/appetite gain vs. loss; psychomotor retardation vs. agitation). This leads to roughly 1,000 unique combinations of symptoms that all qualify for a diagnosis of MDD, some of which do not share a single symptom [5]. It is not surprising that symptom variability among individuals diagnosed with MDD is well-established [5-7].

Cutoff values based on sum-scores from rating scales such as the Beck Depression Inventory (BDI) [8] or the Hamilton Rating Scale for Depression (HRSD) [9] are routinely used as the main criterion to enroll participants in research studies. While the DSM has a hierarchical structure that features two core symptoms, and while symptoms have to cause significant distress or impairment in important areas of functioning for a diagnosis, these criteria are not accounted for in such scales, further increasing the heterogeneity of depressed samples [5].

The next section reviews evidence underlining the importance of attending to particular depression symptoms. We then describe how the use of sum-scores obfuscates important insights in various domains, and suggest that this may help to explain slow progress in key research areas, such as identifying biomarkers and more efficacious antidepressants. We conclude the review with a list of suggestions that have practical research implications.

## Review of symptom-based depression research

Extensive research has described individual depression symptoms; however, the significance of individual symptoms has not been systematically reviewed previously. Here, we describe how attending to specific symptoms has led to insights in research on biomarkers, antidepressant efficacy, depression risk factors, impaired psychological functioning, and causal effects among particular depression symptoms.

### Symptom specificity in biomarker research

Despite extraordinary research expenditures and large genome-wide association studies, no pathognomonic biological markers of depression have been identified. This has been a major disappointment. In 1980, the DSM-III [10] preamble predicted that biomarkers associated with most diagnoses would be identified by the time the DSM-IV [11] appeared; 35 years and two DSM versions later, and with the exception of some neurological disorders, not one biological test for mental disorders was ready for inclusion in the criteria sets for the DSM-5, and not a single psychiatric diagnosis can be validated by laboratory or imaging biomarkers [12].

For depression research, results are specifically disappointing. In a recent large genome-wide association study with 34,549 subjects, no single locus reached genome-wide significance [13]. This is consistent with numerous other large genetic studies that have failed to identify any confirmed associations for MDD [14-17]. Studies predicting antidepressant response by common genetic variants have led to similarly disappointing results [18].

The analysis of specific symptoms offers opportunities to investigate biological factors that may be related to specific syndromes. Jang et al. [19] showed that 14 depression symptoms differ from each other in their degree of heritability (h^2^ range, 0–35%). Somatic symptoms such as loss of appetite and loss of libido, as well as cognitions such as guilt or hopelessness (possibly reflecting heritable personality traits), showed higher heritability coefficients than other symptoms like negative affect or tearfulness. Another study [20] revealed differential associations of symptoms with specific genetic polymorphisms; for example, the symptom ‘middle insomnia’ assessed by the HRSD was correlated with the GGCCGGGC haplotype in the first haplotype block of *TPH1*. In addition, a recent report of 7,500 twins identified three genetic factors that exhibited pronounced differential associations with specific MDD symptoms [21]; the authors concluded that the “*DSM-IV syndrome of MD*[D] *does not reflect a single dimension of genetic liability*” (p. 599). Guintivano and Brown [22] analyzed several independent samples of post-mortem brains and blood samples from living subjects to document that 80% of the variation in one of the most relevant specific symptoms, suicidal behavior, could be explained by how polymorphisms of the gene *SKA2* interacted with anxiety and stress.

Moving away from genes and gene expression to hormones, the hypothesis that depression can be caused by inflammation has received considerable attention in recent years [23,24]. However, evidence shows that less than half of the individuals diagnosed with depression exhibit elevated inflammatory markers [25], and elevated levels of cytokines are neither highly sensitive nor specific to MDD [26]. Furthermore, somatic symptoms such as sleep problems, appetite gain, and weight gain seem elevated in the context of inflammation [27-29], suggesting symptom specificity. A recent review acknowledges intragroup variability of MDD as main limitation of the research on inflammation and depression [26], and suggests that future analyses of distinct endophenotypes may move the field forward.

In summary, individual depression symptoms differ in their biological correlates. This underlines the heterogeneous nature of depression, which may in turn explain the lack of progress in validating depression diagnosis with biomarkers. Analyzing associations between symptom sum-scores and genetic markers can only capture the shared genetic variance of all symptoms, which may be low. A symptom-based approach offers opportunities for future research that could provide a potential partial explanation for the “*mystery of missing heritability*” [30] – the conundrum that specific genetic markers explain only small proportions of the variance even for mental disorders that are highly heritable. Specific markers may correlate better with specific symptoms independent of diagnostic categories – genes do not read the DSM [31]. Studies on symptom-polymorphism associations instead of syndrome-polymorphism associations, similar to the one conducted by Myung et al. [20], may prove insightful.

### The impact of antidepressants on specific symptoms

Several large meta-analyses of clinical trials have demonstrated that antidepressants outperform placebos in less than half of the trials, and that clinically relevant improvements can be documented only for a minority of severely depressed patients [32-34]. Part of the difficulty may be that measuring antidepressant efficacy via sum-scores conceals important effects on specific symptoms [35]. Little research has been conducted on the effect of antidepressants on individual depression symptoms compared to the mountain of literature on specific side effects.

Significant side effects for both tricyclic antidepressants and selective serotonin reuptake inhibitors have prevalence rates of up to 27% in clinical trials [36,37], and common side effects include insomnia, hypersomnia, nervousness, anxiety, agitation, tremor, restlessness, fatigue, somnolence, weight gain or weight loss, increased or decreased appetite, hypertension, sexual dysfunction, dry mouth, constipation, blurred vision, and sweating [38,39] (Table 1). Side effects vary across drugs, and some have more benign effects in specific domains. For instance, certain atypical antidepressants have a superior sexual side effect profile [40], and individuals treated with bupropion and nortriptyline show decreased rates of weight gain [41].

**Table 1:** **Depression symptoms and common antidepressant side effects**

**Symptoms prevalent among depressed patients**	**DSM-5 criterion symptoms**	**Antidepressant side effects**
Depressed mood	**+**	**–**
Anhedonia	**+**	**–**
Feelings of worthlessness	**+**	**–**
Appetite/weight problems	**+**	**+**
Sleep problems	**+**	**+**
Psychomotor problems	**+**	**+**
Fatigue	**+**	**+**
Concentration problems	**+**	**+**
Suicidal ideation	**+**	**+**
Anxiety	**–**	**+**
Sexual dysfunction	**–**	**+**

Curiously, some of the common side effects reported by patients are the very symptoms that are used to measure depression (Table 1). This means that reductions in sum-scores thanks to reduced depression are concealed by increases in sum-scores caused by drug side effects. In addition, the instrument most commonly used in clinical trials is the HRSD which, compared to other depression scales such as the BDI, abounds in somatic symptoms that resemble the side effect profile caused by antidepressant treatment [42].

The presence of particular symptoms has been used to predict treatment response. Sleep problems, for instance, reduce the efficacy of depression treatment [43]; patients with persistent insomnia are more than twice as likely to remain depressed [44], and insomnia can become chronic despite successful resolution of depressive symptoms [45]. Other symptoms also moderate treatment efficacy: anxiety symptoms reduce depression remission rates, successful anxiety treatment prolongs depression remission [46-48], and loss of interest, diminished activity, and inability to make decisions predict poorer antidepressant response [49].

The overlap of antidepressant side effects and depression symptoms provides a compelling reason for analyzing symptoms such as weight problems, sleep problems, or sexual dysfunction separately from sum-scores. A detailed analysis of how different antidepressants influence specific symptoms may improve our ability to determine antidepressant efficacy.

### Risk factor heterogeneity

Risk factors identified for depression include previous episodes of depression [50], demographic variables such as age and sex [51,52], and personality traits such as neuroticism [53]. Statistical models use these and other risk factors to predict the presence or absence of depression.

However, risk factors differ for different symptoms as first demonstrated by Lux and Kendler [54], who analyzed the associations of 25 risk factors on 9 different symptoms in a cross-sectional study of 1,015 individuals. The influence of risk factors differed substantially for different symptoms in a pattern the authors found difficult to reconcile with the general practice of summing symptoms. In another large prospective study, risk factors for depression in medical residents showed strong differential impact on changes of depression symptoms over time [55]. Restricting analyses to a sum-score suggested that women are at greater risk to develop depression during residency, but analyzing individual symptoms revealed that male residents were more likely to experience elevated levels of suicidal ideation under stress, whereas female study participants were more prone to develop increases in sleep, appetite, and concentration problems as well as fatigue.

Adverse life events are well-established risk factors for depression [56], and the depression symptoms individuals experience after a life event seem to depend on the nature of the event. In one experimental study, as well as different cross-sectional and longitudinal investigations of college students and adult samples [57-61], specific types of life events were associated with distinct patterns of depressive symptoms. For instance, after a romantic breakup, individuals mainly experienced depressed mood and feelings of guilt, whereas chronic stress was associated with fatigue and hypersomnia [59].

Overall, risk factors differ substantially for different depressive symptoms, and sum-scores obscure such insights. Studying the etiology of specific depression symptoms may enable the development of personalized prevention that focuses on specific problems and symptoms before they transition into a full-fledged depressive episode.

### MDD symptoms differentially impact on functioning

Most depressed individuals suffer from severe functional impairment in various domains of living such as home life, workplace, or family [2,62]. Their impairment is often long-lasting and equal to that caused by other chronic medical conditions such as diabetes or congestive heart failure [63,64]. The question of whether individual depression symptoms differentially impair psychosocial functioning is thus of great importance.

In a study of 3,703 depressed outpatients, DSM-5 criterion symptoms varied substantially in their associations with impairment [65]. Sad mood explained 20.9% of the explained variance of impaired functioning, but hypersomnia only contributed 0.9%. Symptoms also differed in their impacts across impairment subdomains. For example, interest loss had high impact on social activities, whereas fatigue most severely impacted home management. The overall findings are consistent with an earlier study documenting differential impact of DSM-III criterion symptoms of depression on functioning [66].

While these results require replication in different samples, they offer further evidence for the value of considering depression symptoms separately. Not all symptoms contribute equally to severity ratings, and two individuals with similar sum-scores may suffer from dramatically different levels of impairment.

### Causal associations among symptoms

Measuring depression severity by sum-scores of symptoms ignores a plethora of information pertaining to the intra-individual development of depression, including the power of individual symptoms to cause other symptoms.

Insomnia, for example, leads to psychomotor impairment [67], cognitive impairment [68], fatigue [69], low mood [70], and suicidal ideation or actual suicide [71] – symptoms that closely resemble DSM symptomatic criteria for depression (psychomotor problems; fatigue; diminished ability to think or concentrate, or indecisiveness; suicidal ideation). A meta-analysis of laboratory-based sleep loss studies documented the strength of these effects: sleep-deprived subjects performed 0.87 standard deviations (SD) lower than the control group on psychomotor tasks, 1.55 SD lower on cognitive tasks, and reported mood 3.16 SD lower than the control group. Collapsing over all three measures, performance of sleep-deprived subjects at the 50^th^ percentile in their group was equivalent to subjects at the 9^th^ percentile in the control group [72]. Another recent meta-analysis revealed that psychiatric patients with sleep disturbances are about twice as likely to report suicidal behaviors compared to patients without sleep problems, a finding that generalized across various conditions including MDD, post-traumatic stress disorder (PTSD), and schizophrenia [73].

Hopelessness describes negative expectancies about the future [74]. Although not part of the DSM-5 MDD criteria, it plays a major role in the cognitive triad originally described by Beck [75], performs more strongly than some DSM symptoms in distinguishing depressed from healthy individuals [76], and is assessed in various scales. Numerous studies have confirmed the predictive role of hopelessness for suicidal ideation and suicide [71]. The effects are long-reaching: hopelessness predicted suicidal thoughts, attempts, and actual suicide up to 13 years into the future in a large community sample [77], and was identified as a predictor of suicide among psychiatric patients followed for up to 20 years [78]. The association of hopelessness and suicide generalizes from depressed individuals to patients with other psychiatric conditions [79,80], once more underlining symptom specificity irrespective of a given diagnosis. Hopelessness predicts suicide better than the sum-score from an inventory assessing multiple depressive symptoms [80], and mediates the effect of rumination on suicidal ideation and other depressive symptoms in children and undergraduates [81,82]. In adolescents, rumination predicts the development of subsequent symptoms of depression, bulimia, and substance abuse, while depression and bulimia symptoms in turn predict increases in rumination [82,83]. Symptoms are associated in complex dynamic networks that can form vicious circles which transcend any specific diagnosis, a notion that is also supported by recently developed self-report methods demonstrating complex interactions among symptoms [84,85].

In contrast to longitudinal studies that span months or years, experience sampling methods that allow for the analysis of a large number of timepoints over a comparably short timeframe have consistently revealed short-term associations among depression symptoms (for a review, see [86]). For example, sleep quality predicted affect during the next day in a sample of 621 women, while daytime affect was not related to subsequent night-time sleep quality [70], implying a clear direction of causation. Complementing such group-level analyses with longitudinal idiographic studies is likely to contribute important information. Bringmann et al. [87] documented differences among depressed patients in the way their emotions impacted each other across time; for instance, they found the autoregressive coefficient of rumination to vary substantially across participants – rumination at a given timepoint strongly predicted rumination at the next timepoint for some individuals but not for others. Another study identified heterogeneity in the direction of causation between depression symptoms and physical activity [88]. Overall, a growing chorus of voices advocates the study of inter-individual differences [89-91] which may pave the way towards the development of more personalized treatment approaches. Heterogeneity may also help to resolve controversies about how some symptoms cause others. Sleep deprivation, for instance, has rapid mood-enhancing effects in some depressed patients [92], but other reports suggest that sleep difficulties cause low mood [70].

The notion that symptoms trigger, influence, or maintain other symptoms is widely recognized in clinical practice. A major goal in cognitive therapy is trying to break causal links between different MDD symptoms [75] and approaches like mindfulness-based cognitive therapy suggest that stopping rumination prevents it from causing other depression symptoms [93]. Kim and Ahn [94] demonstrated that causally central depression symptoms (symptoms that trigger many other symptoms) are judged to be more typical symptoms of depression by clinicians, are recalled with greater accuracy than peripheral symptoms, and are more likely to result in an MDD diagnosis. The authors concluded that clinicians think about causal networks of symptoms in ways far more sophisticated than the atheoretical DSM approach of counting symptoms.

### Psychometric evidence

Psychometric techniques such as factor analysis (grouping symptoms) and latent class analysis (grouping individuals) are commonly used to address heterogeneity of MDD. In a more detailed discussion of these methods we draw two general conclusions, both of which support the study of individual symptoms [5].

First, extensive efforts to identify specific forms of treatment effective for specific depression subtypes have been disappointing. There has been little agreement about the number and nature of depression subtypes [95-98], and limited success in identifying external validators for subtypes [99-102]. A recent systematic review that compared the results of 34 factor and latent class analyses concluded that they did not provide evidence for valid subtypes of MDD [95], suggesting the analysis of individual symptoms.

Second, most rating scales for depression are multifactorial and do not measure one underlying factor [103-105]. However, individual symptoms are often at least moderately inter-correlated [106], and the first factor – often a general mood factor or higher-order factor – explains substantially more variance than subsequent factors [103,107]. This means that sum-scores certainly carry information about the general psychopathological load of a particular person, but that the approximation may be fairly rough and that summing symptoms may ignore important information [5,108] (for instance, because MDD symptoms are differentially impairing [65] and because sum-scores do not take into account reciprocal interactions of symptoms [108]).

Applying psychometric tools such as item response theory (IRT) and structural equation modeling (SEM) can yield important insights on the level of individual symptoms because they allow the examination of exact relationships between symptoms and underlying dimensions. One example technique that helps to understand such relations is differential item functioning; a prior study testing for this revealed that different MDD risk factors, such as neuroticism or adverse life events, impact on specific depression symptoms, implying that symptoms are ‘biased’ towards certain risk factors [55]. A second practical application is research on residual dependencies. A major assumption of IRT and SEM models is that the underlying latent variables fully explain the correlation of the manifest indicators. This is rarely the case [109], and especially unlikely in the context of MDD, seeing that symptoms influence each other directly [86,110]. Ignoring such residual dependencies unaccounted for by the latent variables, however, can substantially bias inferences [109,111].

## Practical research implications

Few would defend the notion that depression is a homogeneous, discrete disease. Nonetheless, research on depression generally assigns individuals with diverse symptoms to the same disease category, and the search for potential causes then proceeds as if depression is a distinct disease entity, similar to measles or tuberculosis. This could help to explain the inability to find biomarkers or other external variables that can validate the diagnosis of depression [112-116].

Wide-spread reliance on sum-scores exacerbates the problem. Because depression symptoms are understood as interchangeable indicators of MDD, they are counted instead of being analyzed [54,109]. As we have shown above, however, symptoms are not equivalent, and sum-scores add apples and oranges. As a result, two individuals with equal sum-scores may have clinical conditions whose severities differ drastically. This does not deny the possibility that a central mechanism may switch on multiple aspects of depression in some depressed individuals; that obviously occurs, for instance, as a result of interferon treatment that can cause anhedonia, concentration problems, fatigue, and sleep problems [117]. The analysis of individual symptoms is nonetheless likely to reveal patterns that are currently neglected.

We conclude with a list of practical symptom-based implications that could advance depression research:

i) Analyze each symptom separately
ii) Assess non-DSM symptoms
iii) Distinguish between sub-symptoms
iv) Measure symptoms more objectively
v) Assess symptoms across diagnoses
vi) Improve reliability of assessment
vii) Use multiple scales to assess symptoms
viii) Investigate networks of symptom interactions
ix) Investigate symptom profiles in clinical trials

### Improved measurement of MDD symptoms

The first group of research implications is for the measurement of depression symptoms. After reviewing many depression rating scales, Snaith [42] concluded that “*The measurement of ‘depression’ is as confused as the basic construct of the state itself*” (p. 296). Below we explain why this is the case, and suggest several important steps that could reduce confusion.

#### Assessment of important non-DSM symptoms

First, expanding the range of symptoms analyzed may offer new insights. Today’s DSM MDD criterion symptoms were determined largely by clinical consensus instead of empirical evidence – one of the first proposed sets of symptoms goes back to the 1957 report by Cassidy [118], who described clinical features of manic-depressive disorders. The list was reworked later by Feighner [119], without published data to support the changes. Today’s criterion symptoms for MDD closely resemble the ones proposed over 40 years ago, and numerous critical calls for a psychometric (re)evaluation of depression and its symptoms have had little impact (e.g., [54,76,120]). Anxiety and anger are especially interesting symptoms for depression research; both are highly prevalent in depressed patients and associated with worse clinical outcomes [46,121]. In a large clinical trial, over half of the depressed patients reported significant levels of anxiety, and remission of depression was less likely and also took longer in this group [46]. Elevated baseline anxiety levels in treatment studies predict higher depression levels later on [122], and anxiety was identified as a risk symptom for adverse mental health trajectories in a large epidemiological study [123]. Anger is also prevalent among depressed patients, and has been identified as a clinical marker of a more severe, chronic, and complex depression [121]. The recently published Symptoms of Depression Questionnaire includes a variety of non-DSM symptoms, such as anger and anxiety, and may prove an important tool for future research [124].

#### Distinguishing between sub-symptoms

Making more detailed assessments of compound symptoms offers additional opportunities. Insomnia and hypersomnia are opposites; subsuming them into ‘sleep problems’ hampers progress. A recent meta-analysis revealed that the specific sleep problems of insomnia, parasomnia, and sleep-related breathing disorders, but not hypersomnia were related to suicidal behavior across a broad range of psychiatric conditions such as MDD, PTSD, and schizophrenia. Nightmares could also be included in future depression questionnaires, seeing that individuals suffering from nightmares showed a drastically elevated risk for suicidality [125]. Psychomotor problems pose yet another example, the impact of psychomotor retardation on impairment of psychosocial functioning in the Sequenced Alternatives to Relieve Depression (STAR*D) study was four times greater than the impact of psychomotor agitation [65]. Fatigue and sleepiness also need differentiation. As Ferentinos et al. [69] point out, “*insomnia causes fatigue, while sleep apnea and narcolepsy cause mostly daytime sleepiness; fatigue is alleviated by rest, while sleepiness is relieved by sleep* […]. *Unfortunately, however, fatigue and sleepiness may sometimes be confounded in clinical practice, research, and psychometry*” (p. 38).

#### Precise measurement of symptoms

The assessment of symptoms with higher precision offers further opportunities. More complex constructs, such as sadness, could be assessed with more than one question. Self-report information can be augmented with objective data. Patient reports about sleep quality can be complemented by physiological data on sleep patterns and sleep duration. Diaries can track sleep quality and weight changes, and impaired concentration can be measured using tests such as the d2 Test of Attention [126].

#### Transdiagnostic assessment of symptoms

Many symptoms are present in multiple disorders. Mental disorders, such as MDD, PTSD, or generalized anxiety disorder, are highly comorbid [127] in part because they share defining symptoms such as sleep problems. Anxiety is prevalent among many psychiatric conditions. Fatigue is a diagnostic criterion for several DSM disorders, but it also arises from many other medical conditions in ways that can artificially increase depression rates in such populations [128]. These symptoms may thus not be particularly useful for determining the presence of depression. However, the transdiagnostic study of common psychopathological symptoms – e.g., the similarities and differences of fatigue across different conditions – may offer substantial insights.

This idea also has implications for semi-structured interviews, such as the Structural Clinical Interview for DSM Disorders (SCID). In contrast to most scales, these instruments offer the opportunity to assess a large amount of symptoms from different diagnoses. However, it is currently impossible to utilize data gathered via semi-structured interviews for symptom-based research due to the skip questions. Skip questions are a heuristic to save time both for the interviewer and the interviewee: if an individual reports none of the core symptoms necessary for a diagnosis (such as anhedonia and sad mood for MDD), all other symptoms are skipped. While this speeds assessments, it loses vast amounts of information about specific symptoms. Researchers employing the SCID and similar instruments who query study participants about all symptoms even in the absence of core symptoms will generate important new findings.

#### Reliability of symptom measurement

One of the main challenges for symptom-based research is reliably measuring symptoms. Common rating scales were often not designed or validated for using symptom-level information. Instead, the assessment of symptoms was meant as measurement for an underlying disease [109]. This is an advantage of sum-scores: they include a number of at least moderately correlated symptoms, and are thus less susceptible to this measurement problem.

A possible solution to increase the reliability of symptom assessment for self-report questionnaires or clinical interviews is to follow the general psychometric practice of assessing variables of interest with more than one item. A good example is the Inventory of Depression and Anxiety Symptoms that uses multiple questions per symptom domain. For instance, suicidal tendencies are measured via 6 different items [129], allowing for a more reliable measurement. If this became standard practice, it would likely reduce measurement error on the symptom level.

#### Use of multiple depression scales

Finally, for studies that must rely on symptom sum-scores, different depression instruments should be utilized simultaneously, and conclusions should be considered robust only if they generalize across different scales. Despite their aim to measure the same underlying construct, there are marked differences between different instruments for measuring depression. For instance, scales differ in how they classify depressed patients into severity groups, so the scale chosen for a particular study can bias who qualifies for enrollment, and who achieves remission [130]. Instruments also include a variety of different symptoms, and their sum-scores are often only moderately correlated, suggesting that results may often be idiosyncratic to the particular scale used in a study [42,103,104,131]. In a review of 280 different depression scales, Santor et al. [131] concluded that most research is based on just a few scales, such as the HRSD and BDI, so much of what we know about depression depends on the quality of these scales. This is bad news, considering the low psychometric quality of the HRSD and BDI (poor inter-rater reliability, poor re-test reliability, poor content validity, and poor psychometric performance of certain items) [104,105]. While some changes were made to the DSM criteria in the last decades, most rating scales used today are at least 20 years old (in the case of the HRSD, half a century) and do not reflect these changes; most do not even include all nine DSM-5 criterion symptoms [103].

### Network models

While the more traditional SEM and IRT models assume that all depression symptoms share a common cause and are locally independent (i.e., uncorrelated beyond the common cause; see [109]), a growing number of studies have shown that symptoms can trigger other symptoms. A recently developed framework – the network approach to psychopathology – allows the study of such dynamic interactions. Network models estimate the relationships among symptoms within or across time [106,109,110], and offer a new perspective on why symptoms cluster. While latent variable models explain symptom covariation by a latent factor that is viewed as the common cause of all symptoms, network models suggest that syndromes are constituted by the connections among symptoms. This perspective encourages consideration of how vicious circles of symptoms can fuel each other, an alternative to the schema in which all symptoms arise from a single brain disorder.

### Reporting of symptom profiles

We anticipate fundamental advances from researchers who report and analyze information about specific symptoms. For instance, inconsistent reports about the efficacy of antidepressants may result from samples with different symptom patterns that may respond differently to different agents. A meta-analysis to test this hypothesis requires data on individual symptoms that is not available in the Food and Drug Administration database of depression studies.

A recent study by Uher et al. [132] suggests the available opportunities. The authors found that individuals with high baseline levels of systemic inflammation exhibited increased depression recovery under nortriptyline, while low inflammation levels were associated with superior depression improvement under escitalopram, supporting earlier work on the topic [133]. These results are especially interesting considering that inflammation levels are particularly elevated among depressed individuals with somatic symptoms [28], specifically appetite and weight gain [27]. If patients with high and low baseline inflammation levels exhibit different symptoms, it should be possible to select study participants who will respond to a particular drug. Finding biological markers for specific depressive symptoms will open new research vistas.

## Conclusions

Depression symptoms are commonly added up to create sum-scores that are assumed to reflect the severity of a uniform underlying depressive disorder. This schema discards data about specific symptoms, treating all as equivalent and interchangeable indicators of MDD. It also fosters asking simplistic questions such as ‘what causes depression?’ or ‘what treatment is best for depression?’ Analyzing specific symptoms and their causal associations is an initial step towards personalized treatment of depression that recognizes the heterogeneity of MDD. This is certainly more complicated than the study of sum-scores, but well worth the effort. As John Tukey [134] pointed out, “*Clarity in the large comes from clarity in the medium scale; clarity in the medium scale comes from clarity in the small. Clarity always comes with difficulty*” (p. 88).

## Abbreviations

BDI: Beck Depression Inventory
DSM: Diagnostic and Statistical Manual of Mental Disorders
HRSD: Hamilton Rating Scale for Depression
IRT: Item response theory
MDD: Major depressive disorder
PTSD: Post-traumatic stress disorder
STAR*D: Sequenced Alternatives to Relieve Depression
SCID: Structural Clinical Interview for DSM Disorders
SEM: Structural equation modeling
